# Automating image-based mesh generation and manipulation tasks in cardiac modeling workflows using Meshtool

**DOI:** 10.1016/j.softx.2020.100454

**Published:** 2020-03-20

**Authors:** Aurel Neic, Matthias A.F. Gsell, Elias Karabelas, Anton J. Prassl, Gernot Plank

**Affiliations:** a Gottfried Schatz Research Center: Division of Biophysics, Medical University of Graz, Graz, Austria; b Department of Biomedical Engineering, School of Biomedical Engineering & Imaging Sciences, King’s College London, London, United Kingdom; c NumeriCor GmbH, Graz, Austria

**Keywords:** Mesh manipulation, Geometric smoothing, Mesh generation

## Abstract

Advanced cardiac modeling studies rely on the ability to generate and functionalize personalized *in silico* models from tomographic multi-label image stacks. Eventually, this is used for building virtual cohorts that capture the variability in size, shape, and morphology of individual hearts. Typical modeling workflows involve a multitude of interactive mesh manipulation steps, rendering model generation expensive. Meshtool is software specifically designed for automating all complex mesh manipulation tasks emerging in such workflows by implementing algorithms for tasks describable as operations on label fields and/or geometric features. We illustrate how Meshtool increases efficiency and reduces costs by offering an automatable, high performance mesh manipulation toolbox.

## 1 Motivation and significance

**Fig. 1:**
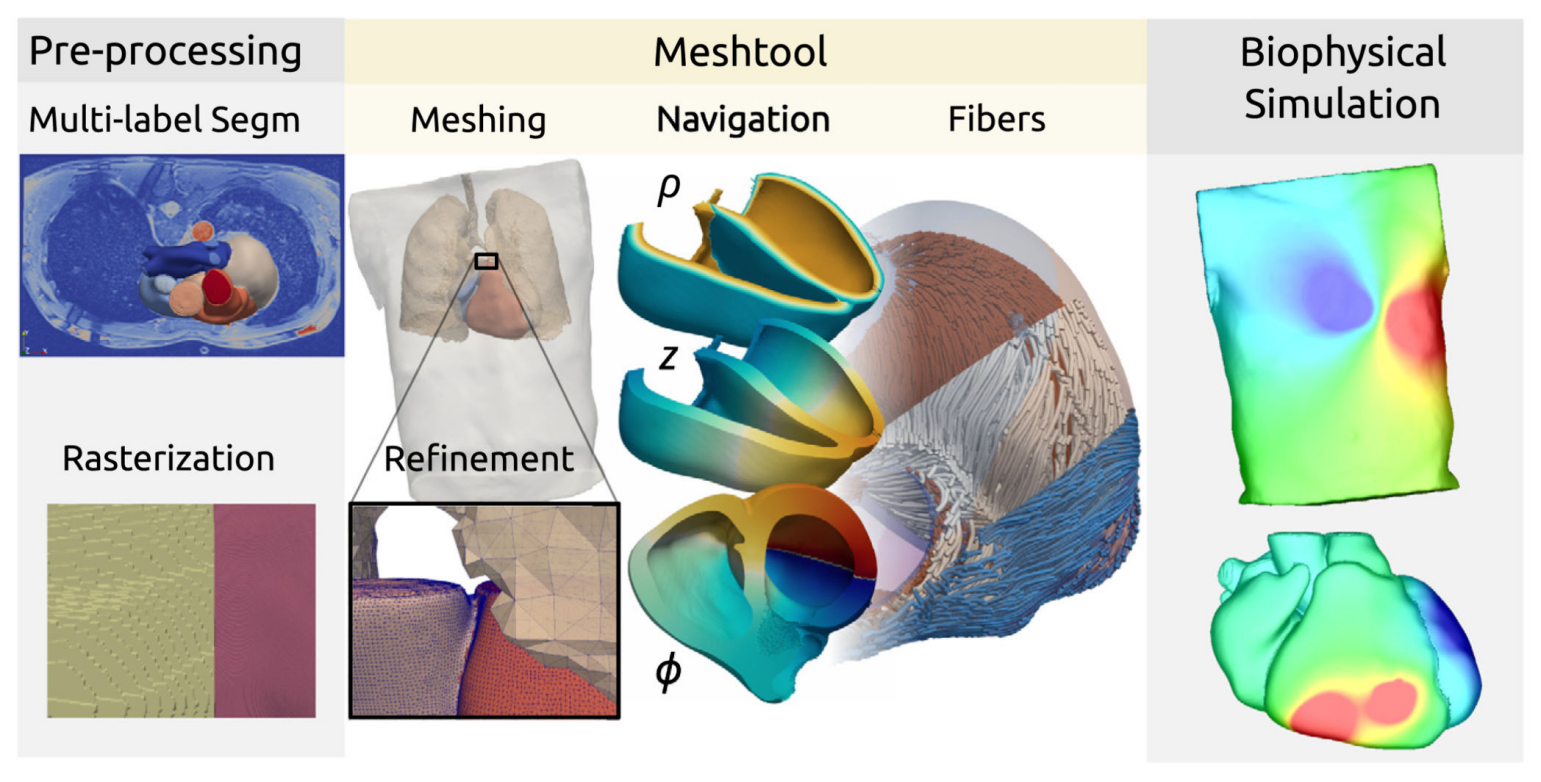
Typical model building and functionalization workflow showing the processing stages of multi-label segmentation, re-rasterization to higher isotropic resolution, meshing and label transfer, definition of boundary conditions, computation of a coordinate system for navigation, assignment of structural properties such as fiber and sheet arrangements and mesh refinement for carrying out biophysical simulation.

The development of Meshtool was motivated by demands of modern cardiac modeling applications. These pose a set of specific challenges on meshing technology for both the representation of cardiac anatomy as well as for the functionalization of the biophysical models used to describe cardiac behavior. The workflows for generating advanced *in silico* models consist of numerous processing stages, turning medical images into discrete mesh-based representations suitable for solving physical problems, see Fig. 1. Generation of meshes from segmented medical images has been identified as a key enabling technology in the chain of workflows. Many advanced software solutions, like Tetgen [1], CGAL [2], or ANSYS^®^ Meshing,^1^ have been under constant development in both industry and academia. However, these were mostly geared towards regular engineering applications where the geometry of objects is represented by CAD-constructed surfaces, and to a much lesser extent, towards image-based meshing application where geometric and anatomical information is based on volumetric data encoded in segmented multi-label image stacks [3].

An additional specific demand in cardiac modeling stems from the multiphysics nature of cardiac function. Depending on which aspect of cardiac function is under investigation, be it electro-physiology, mechanics, hemodynamics or perfusion, the requirements for spatial discretization vary markedly with resolutions ranging from 1 mm to 100 μm. Therefore, in an initial meshing step, the spatial resolution shall be chosen to allow a sufficiently accurate – within the limits of uncertainty of segmentation of clinical input data – and smooth representation of the organ. Label fields, which encode the membership to specific cardiac structures, will then be transferred onto the mesh [4]. Any physics or problem-specific adjustments shall be carried out subsequently, on demand when needed, in a fully automated fashion to meet the requirements of the physical problem being solved.

For the sake of model functionalization any current cardiac multiphysics modeling study relies on the ability to extract sub-meshes, including corresponding label fields as well as scalar, vector-valued or tensor-valued data defined on a given sub-mesh, carry out operations on extracted sub-meshes, and reinsert sub-meshes and manipulated data back into a reference mesh.

A recently emerging trend is the use of cardiac models in industrial application such as the design of cardiac devices for the optimization of device-based therapies. Such applications, labeled as medical device development tools (MDDT) by regulatory bodies, require the fusion of descriptions of device geometries, typically defined as CAD surface models, with volumetric meshes of heart and torso. The stimulation electrodes of cardiac devices such as pacemakers are implanted in specific domains of the heart, usually in the blood-filled cavities, in vessels or subcutaneously within the torso. Considering the cost of the entire model generation workflow – from multi-label image data sets to multidomain meshes – the ability to integrate CAD-based device descriptions with pre-existing meshes of the heart without re-meshing all domains is highly advantageous.

Moreover, for capturing biological variability, current studies attempt to build *virtual cohorts* consisting of larger number of models. Modeling applications of this high throughput critically depend on a high degree of automation, as the use of interactive meshing environments is too tedious, time-consuming and expensive in terms of man power needed. Departing from a well defined image-based geometric description of a heart where all anatomical structures are classified in the form of label fields, a baseline mesh at a given target resolution can be generated without any need for operator interaction in a fully automated fashion. As such, all further downstream manipulations of the baseline mesh, such as resolution adjustments or the integration of physics-specific data fields, should also be automated and controlled abstractly based on label field operations.

The software Meshtool was developed to meet these specific demands of cardiac modeling applications. It offers all capabilities needed in pre-processing workflows – such as uniform mesh refinement, mesh re-sampling, mesh and data extraction and insertion, local re-meshing for geometry integration and the ability to be fully automated – with solid performance characteristics and robust problem size scaling. This renders it a versatile tool for automating anatomical model building and functionalization workflows [4]. Having this ability is instrumental in currently emerging *in silico* modeling studies where a large number of models [5,6] – on the order of tens to hundreds – have to be constructed and functionalized by fitting model predictions to observed data [7,8]. Being able to generate large virtual cohorts is poised to leverage *in silico* modeling as a medical device development tool (MDDT) [9] and render advanced clinical application feasible where modeling software is used as a medical device (SaMD) [5,6]. Meshtool has been adopted quickly within our collaborative network which led to a number of publication over a broad range of applications [9–11].

## 2 Software description

### 2.1 Software architecture

Meshtool is written in C_++_(2011 standard support required). It has no external library dependencies, which leads to a very simple compilation process. The only required third-party code is the open-source tetrahedral mesh generation library Tetgen, which is used for partial re-meshing. The Tetgen library has been integrated into the Meshtool repository and its compilation process. This streamlines the overall compilation process, simplifies code distribution and improves reproducibility of the software builds.

The base of the software is formed by a set of structures and classes that hold the computational data. Contiguous blocks of data are stored with the templated mt_vector class, which is designed similarly to the std::vector container of the standard C_++_library. Most higher-level containers, such as mesh, graph and mapping classes, use mt_vector for storing internal data. Accelerated data lookup is either implemented by the C_++_standard library std::set and std::map containers, or, when ordering is not relevant, by custom hashmap::unordered_set and hashmap::unordered_map implementations.

The core of the software is formed by the different utility classes and functions. Essentially, these algorithms represent the main building blocks from which the Meshtool modes are built. Algorithms that hold a state through multiple computation steps are mostly implemented as classes. Examples for such algorithms are the edge splitting and collapsing algorithms in mt_edge_collapser and mt_edge_splitter, which hold the edge list and other state variables between multiple re-sampling operations. On the other hand, state-less algorithms are implemented as functions. Many algorithms use graphs derived from the mesh connectivity for mesh traversal.

The top layer is formed by the different Meshtool modes, each implemented in an individual source file. Each mode is designed as a self-contained unit, that could also be an own executable. In fact, some modes that are deemed too specific and not general enough to be part of Meshtool, are compiled as separate executables in the *standalones* subdirectory.

**Fig. 2:**
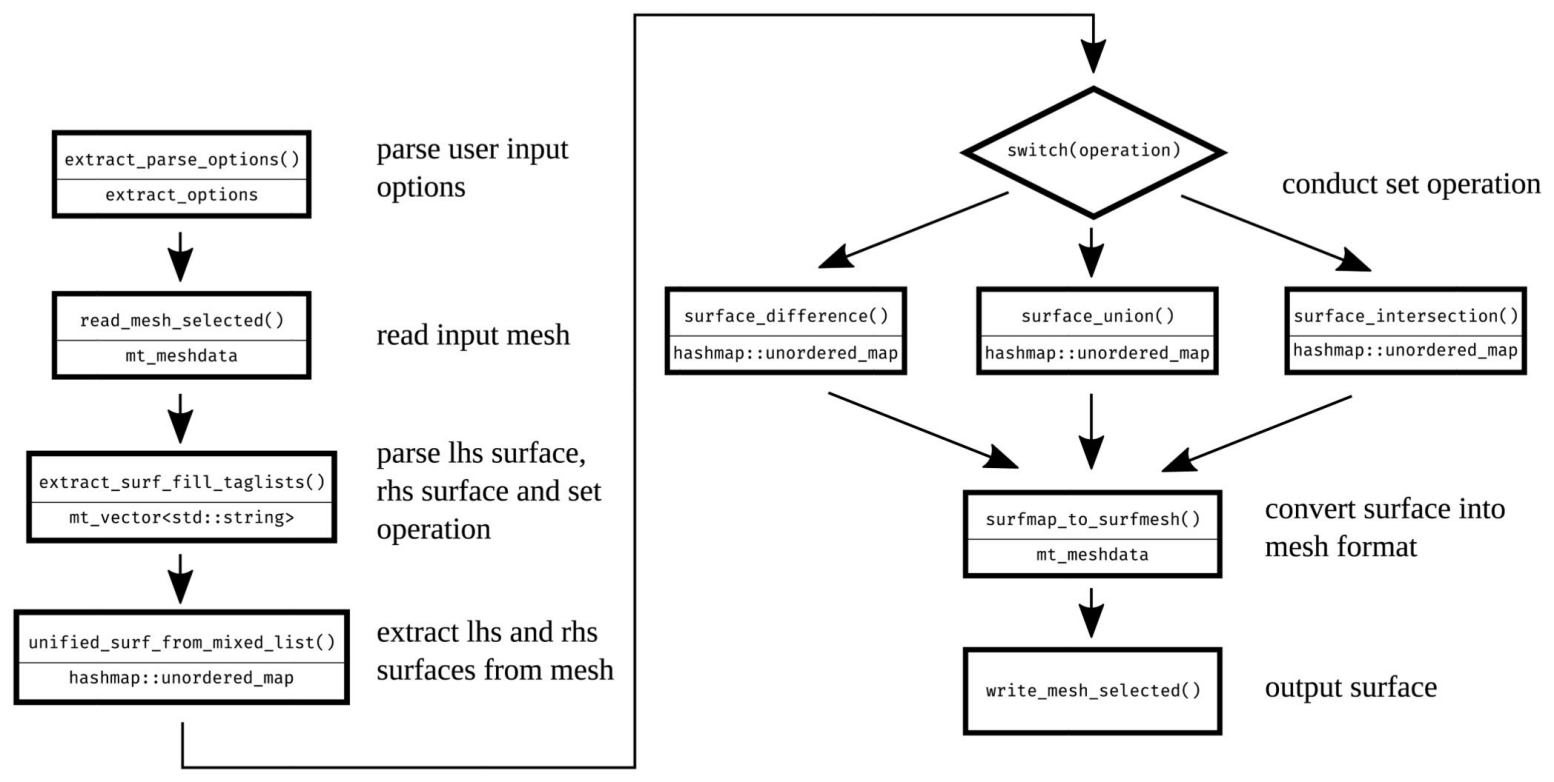
Flowchart of a surface extraction operation. Each processing step displays its function name in the upper line and the key output data-structure in the lower line.

Fig. 2 gives an example of a Meshtool mode architecture, by depicting the flowchart, with the main data-structures and functions, used for surface extractions.

### 2.2 User interface

Meshtool is operated through a command-line interface (CLI) and can be used in a completely automated manner. Still, it provides the user with progress outputs and time-to completion estimates when used interactively.

In order to improve usability, the CLI interface is structured similarly to those of the *git* version control system or the Linux package management tool *apt*: The user calls Meshtool with the desired *mode* as the first program parameter, before setting mode-specific options with the subsequent parameters. As such, the set of options the user is exposed to is reduced to only those relevant to the selected mode.

Many modes consist of two words, usually a verb and an object, for example extract surface or interpolate nodedata. If the user specifies only the first word of a two word mode, Meshtool prints all modes starting with the first word. Calling a Meshtool mode with none or insufficient options, displays the mode-specific options in a help message. This design allows the user to briefly query the availability and usage of different modes without having to memorize help-specific options. Still, Meshtool also offers a help mode that lists all available modes along with a short description. The most important modes are listed in Table 1.

### 2.3 Software functionalities

The Meshtool utility consists of an agglomeration of currently over forty modes, tuned for optimal inter-operability and automatibility. Rather than presenting all of them, we restrict ourselves to a small subset that we deem representative. We want to emphasize, that the goal of Meshtool is to provide users with a wide variety of mesh manipulation tools, that can be combined and automated in a consistent manner. We do not claim that any single tool represents the cutting edge in its category and therefore refrain from direct comparisons to the state-of-the-art.

#### Set operation based surface extraction

In cardiac electrophysiology mesh generation, landmark surfaces are defined as the set intersection of specific anatomical regions. For example, the endocardial surface can be extracted by computing the set intersection of the ventricular tissue and the enclosed blood pool. In an automated mesh generation workflow the labeling of specific regions is standardized to facilitate the automated extraction of all landmark surfaces of interest such as epicardium, endocardium, apex or basal plane.

The Meshtool mesh extraction mode allows to compute set union, intersection and difference on surfaces. A surface can be defined by the user in two ways: Either by a set of region labels, which define a submesh boundary surface, or directly with a surface file.

**Fig. 3:**
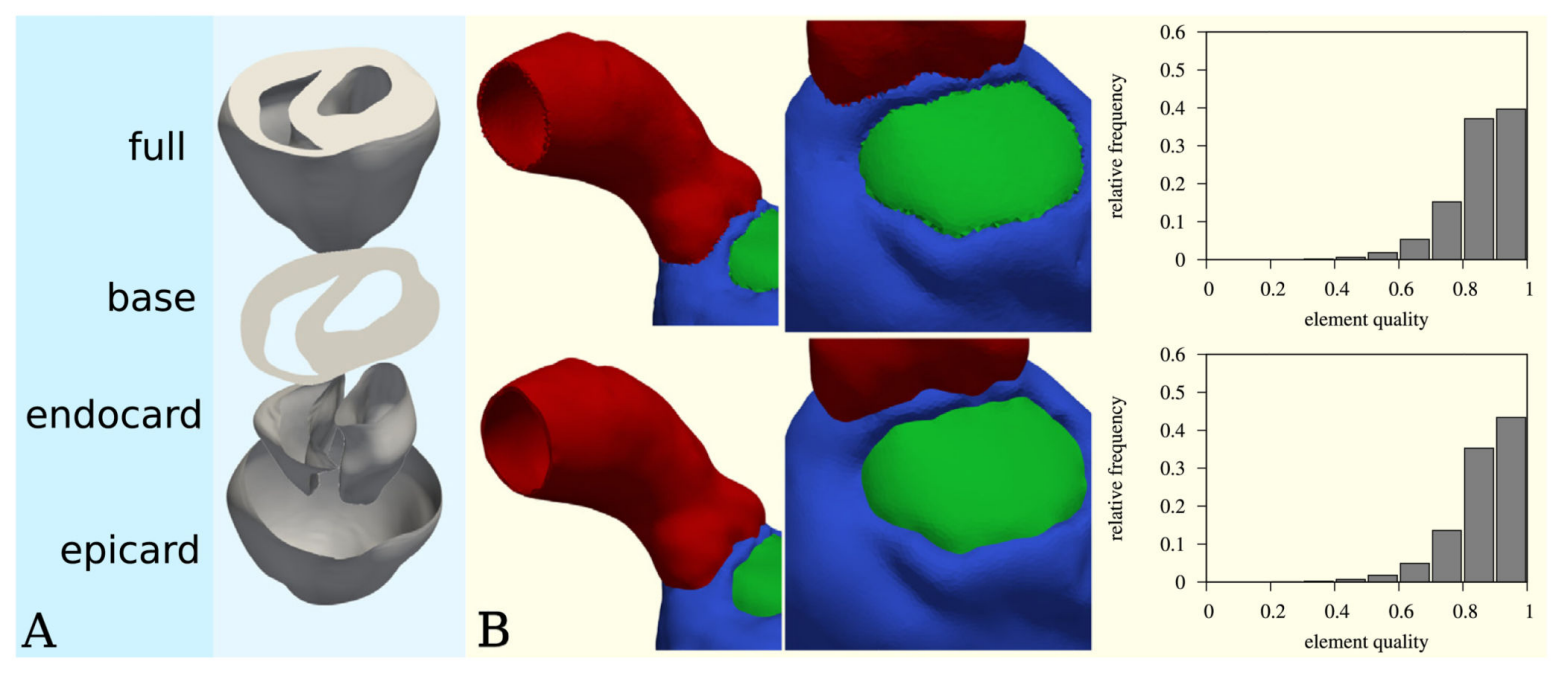
Examples for mesh smoothing and surface extraction. (**A**) The epicardium, endocardium and cardiac base surfaces are extracted from a rabbit ventricular heart model using set intersection and traversal from seed points with blocking sharp edges. (**B**) A volumetric mesh is smoothed to achieve smooth interfaces between the individual mesh regions. The top images show the mesh before smoothing, the bottom images show the mesh after 200 smoothing iterations. The right column shows histograms of the element quality before (top) and after (bottom) smoothing. The used quality metric was the volume-edge ratio, with the quality 0 representing the worst and 1 the best element quality.

The surface resulting from the set operation can be further restricted to areas accessible via edge traversal from chosen seed locations. Edge traversal can be further configured through options such as traversal distance or the definition of a critical curvature of edges above which further traversal is blocked. Fig. 3A shows an example of epicardial, endocardial and base surface extraction on a rabbit heart of ≈ 5000k elements and ≈ 800k vertices. Each surface extraction required about two seconds on a workstation computer.

#### Shrinkage-free mesh and data smoothing

A common observation when applying a basic smoothing scheme like Gaussian smoothing to a data-set, be it mesh vertex coordinates, scalar data or vector valued data, is that the entries in the data set converge towards the global average with each successive smoothing iteration. In the context of mesh surfaces, this effect is observed as mesh volume shrinkage.

In order to counter this effect, shrinkage-free smoothing algorithms have been formulated [12,13]. The central idea is to move towards the local average in one iteration, and then move away from the local average in the next. The overall effect is that of a low-pass filter and as such, mesh volume is preserved. The Mesh-tool modes smooth mesh, smooth surface and smooth data use Taubin’s method [12] to apply low-pass filters to meshes and/or data.

When meshes are smoothed, Meshtool decomposes the volumetric domain, if applicable, into volumetric, surface and line manifolds. Average positions for a vertex are computed only with respect to the vertices in its respective manifold set. This approach is vital for achieving smooth surface and line interfaces when smoothing volumetric meshes.

The implemented mesh smoother can also track the mesh quality. In essence, smoothing updates are only applied as long as a quality threshold is not crossed. This allows for robust application of mesh smoothing without risking mesht quality deterioration beyond the specified threshold. Currently mesh quality tracking is only supported for tetrahedral meshes. The implemented quality metrics are volume-edge ratio [14] and minimal dihedral sine [15]. For more details we refer to [16].

Fig. 3B shows an example of how smooth interfaces between different mesh regions can be achieved by volumetric smoothing considering surface and line manifolds. The depicted left-ventricle mesh consist of ≈ 2500k elements and ≈ 400k vertices. The smoothing was performed in fifteen seconds on a desktop workstation. The mesh quality histograms indicate that quality-aware smoothing has a positive effect on mesh quality, while also smoothing the geometry.

#### Mesh re-sampling

Meshes consisting of triangles or tetrahedra can be re-sampled to different resolutions using the resample mesh and resample surface modes. The user specifies the minimum, maximum or average edge lengths desired for the re-sampled target mesh. The re-sampling algorithm then uses iterative edge-bisection [17] and edge collapsing algorithms [18] to change mesh resolution to the targeted range.

The re-sampling algorithm consists of two main steps: In the first step, edges longer than the maximum size threshold are split iteratively. In the second step, edges smaller than the specified minimum size threshold are iteratively collapsed. Some edges may not be collapse-able due to quality considerations and as such the minimum size threshold may not be enforceable on all edges.

When collapsing edges, the two main challenges are the preservation of mesh quality and shape. Mesh quality is preserved by dismissing collapse attempts that would result in intersections of elements or degenerated elements. Mesh shape preservation can be controlled through a user-set parameter that sets a threshold on the acceptable surface normal change during edge collapse.

**Fig. 4:**
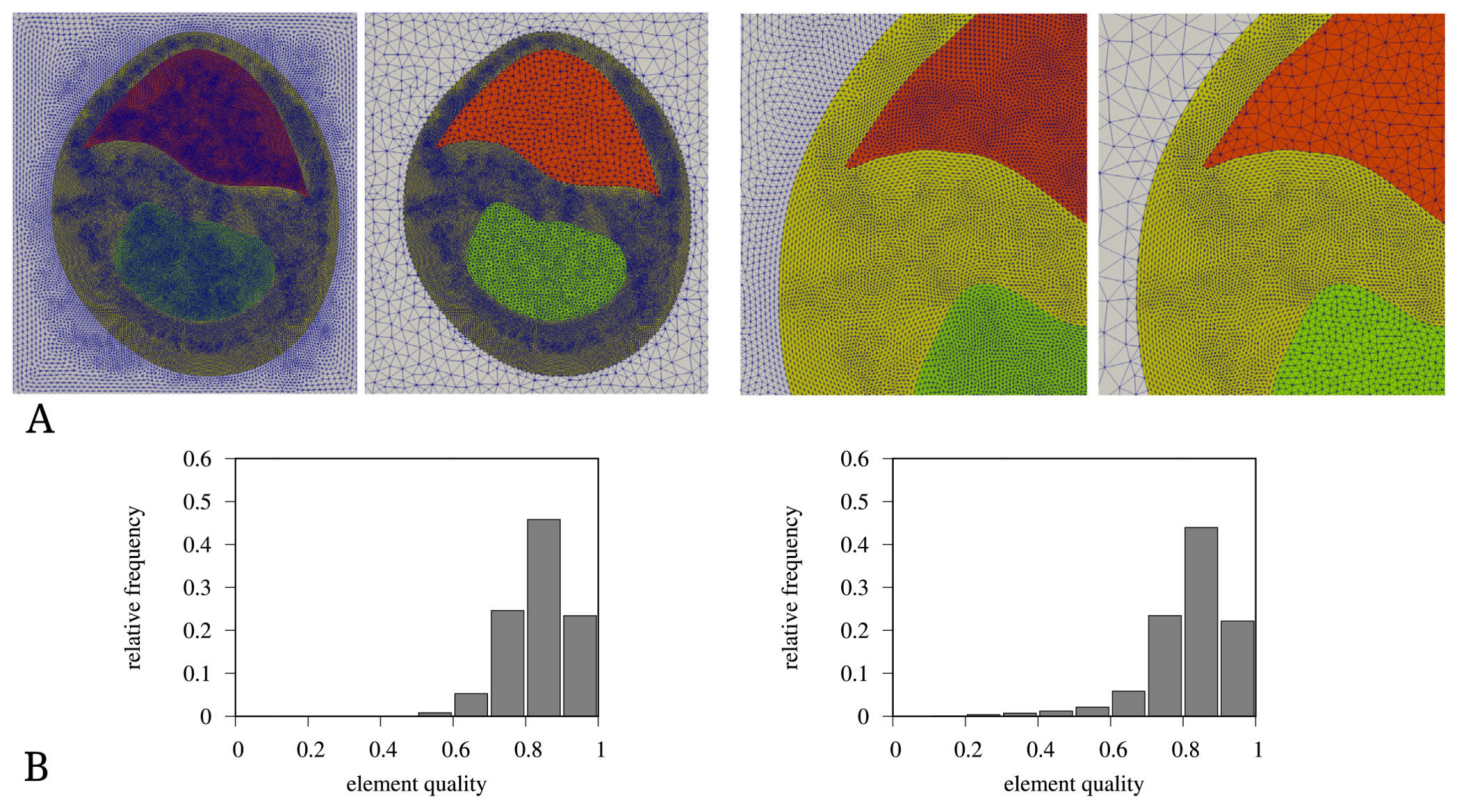
Re-sampling examples of a rabbit biventricular slice mesh. (**A**) Three regions of a mesh are re-sampled to different average resolutions. The white region is down-sampled from a mean resolution of 350 μm to 750 μm, the red region from 180 μm to 500 μm and the green region from 180 um to 300 μm. (**B**) Histograms of the element quality before (left) and after (right) re-sampling. The used quality metric was the volume-edge ratio, with the quality 0 representing the worst and 1 the best element quality.

For meshes with tagged element regions, Meshtool allows to restrict the re-sampling to sets of region tags. Fig. 4 shows different re-sampling operations on a rabbit heart slice mesh of ≈ 500k elements and ≈ 110k vertices. The re-sampling operations required between seven and thirteen seconds on a desktop workstation. The element quality histograms indicate that overall mesh quality was slightly reduced by the re-sampling.

## 3 Illustrative examples

**Fig. 5:**
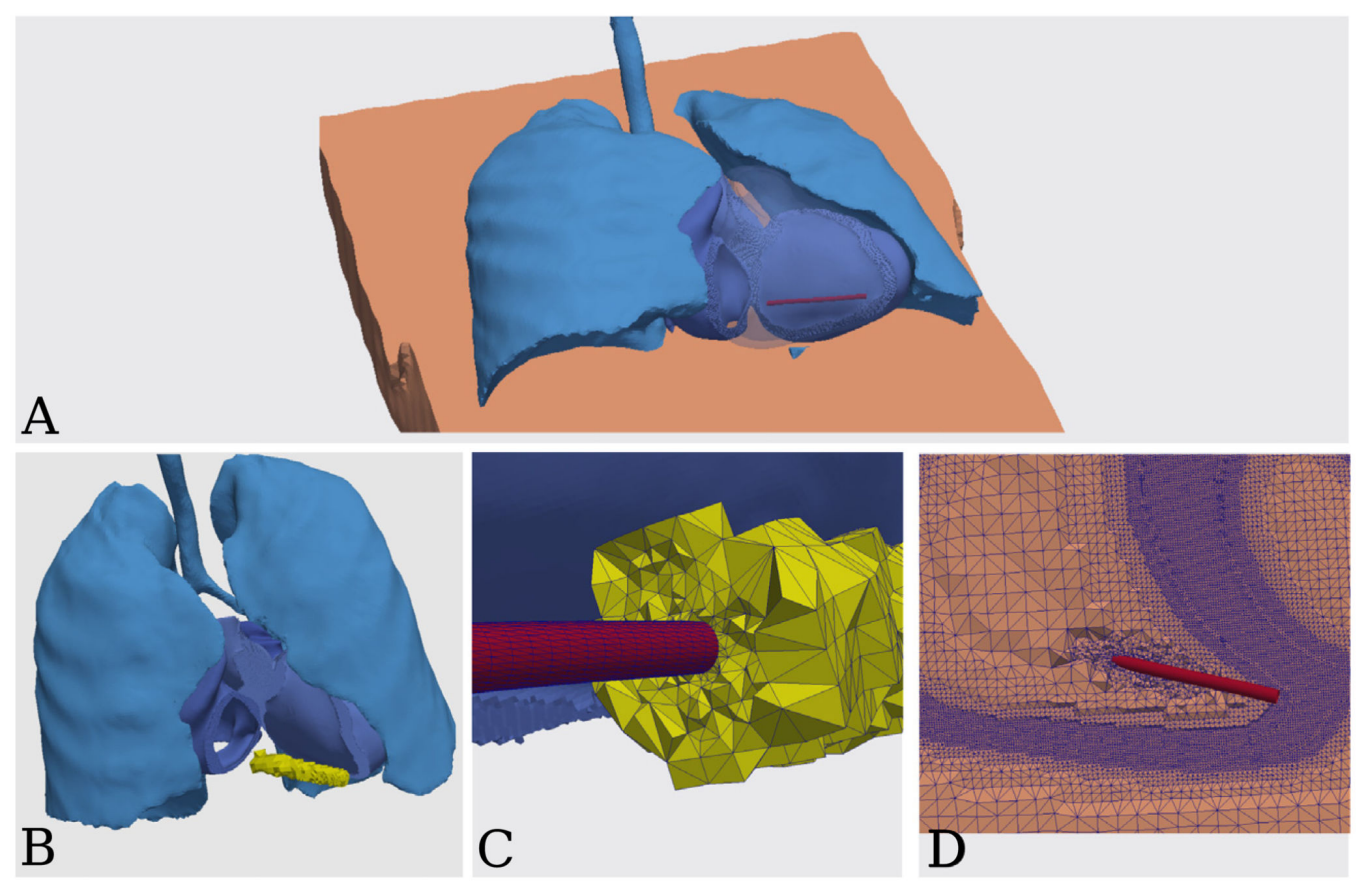
Medical device integration example. (**A**) The coil (in red) placement inside the right ventricle blood pool. (**B**) Extraction of the blood pool volume (in yellow) overlapping with the coil geometry. (**C**) Clip through the re-meshed blood pool and coil volumes. (**D**) Clip through the final torso with medical device mesh volume. (For interpretation of the references to color in this figure legend, the reader is referred to the web version of this article.)

In this example we integrate a defibrillation coil surface geometry originating from a CAD program (≈ 18k elements and ≈ 9k vertices), into an existing MRI-based geometry of a human torso with segmented lungs and heart (≈ 48000k elements and ≈ 8000k vertices), see Fig. 5.

The example makes use of the sub-mesh extraction, surface extraction, re-meshing and mesh merging modes of Meshtool. The input data is a torso mesh and the coil surface geometry, already transformed into the desired location in the right-ventricle blood pool. 
TORSO=torso.vtk
COIL=coil.rv.vtk



In the first step, we extract the volume blood pool volume overlapping with the coil. Meshtool outputs the overlap torso.ovlp.vtk, depicted in Fig. 5B and its complement to the full mesh torso.ovlp.compl.vtk. 
meshtool extract overlap -msh1=$TORSO -msh2=$COIL \
-submsh=torso.ovlp.vtk -mode=1 -size=3.0
 We want to re-mesh the extracted overlap volume, such that it includes the coil geometry. This is done by passing the surface meshes of the bloodpool overlap and the coil to the generate mesh mode of Meshtool.

Therefore, we next extract the surface of the overlap. If a mesh output format is specified with the option -ofmt, Meshtool will not only generate a surface definition (i.e. a list of element faces) in torso.ovlp.surf, but also a surface triangle mesh torso.ovlp.surfmesh.vtk. 
meshtool extract surface -msh=torso.ovlp.vtk \
-surf=torso.ovlp -ofmt=vtk_bin
 Now we call the mesh generation mode. With the -ins_tag we define the region IDs for the elements inside each input surface. The result is depicted in Fig. 5C. 
BLOODPOOL_TAG=90
RV_COIL_TAG=502
meshtool generate mesh -surf
  =torso.ovlp.surfmesh.vtk,$COIL \
-ins_tag=$BLOODPOOL_TAG,$RV_COIL_TAG -outmsh
  =rv.meshed.vtk -scale=1.8
 In the final step we insert the newly meshed volume into the complement to the input torso mesh. The complement was generated in the initial overlap extraction. 
meshtool merge meshes -msh1=torso.ovlp.compl.vtk \
-msh2=rv.meshed.vtk -outmsh=torso.final.vtk



This example executed in 2 min and 30 s on a desktop workstation.

## 4 Impact

The ability to generate, in a highly automated fashion, anatomical models of high geometric fidelity from labeled image stacks, and functionalize these with appropriate parameter fields, is of major importance in cardiac modeling endeavors. Advanced studies rely on virtual cohorts comprising a larger number of *in silico* models which match anatomy and function of their *in vivo* counterparts with high fidelity. Applied research using such models has been hampered in the past by the high costs involved. A large number of processing steps must be executed involving interactive mesh manipulation procedures of expert users at each stage. Meshtool provides all necessary mesh manipulation algorithms which are needed to generate anatomy models and assign all geometry-dependent parameter fields in a fully automated fashion. It should be noted, that this ability relies heavily on the detailed classification of voxels in the input image stack as all operations must be abstractly describable based on labels and/or geometric features. Depending on the type of model and the level of geometric detail required for a particular application, the segmentation and labeling of the input meshes may constitute a challenge on its own [19].

The integration of Meshtool constitutes a step change in the ability of our lab to generate geometrically accurate functionalized *in silico* models of individual hearts. Due to the anatomical variability between individuals, any cardiac modeling study that aims to investigate mechanisms of cardiac function in a broader sense, depends critically on the availability of larger virtual populations that cover this variability. A software such as Meshtool is indispensable in efficient large-scale cardiac modeling endeavors as it turns model generation from an expensive procedure requiring days of attention of highly trained expert users into a basic commodity that achieves these goals in an inexpensive manner and can be operated by non-expert users with ease. The Meshtool-based automation of the model building pipelines also led to a noticeable reduction in model errors by avoiding any error-prone interactive manipulations. This improved model quality and avoided the costs of failure at the later simulation stages due to faulty meshes introduced by manual mesh manipulation. In such cases the manipulation step causing the problem needed to be identified and corrected. Owing to dependency of the building pipelines, all subsequent processing steps had to be repeated, again involving manual work of operators. The corrected meshes which are, typically, of size at the order of gigabytes of data, had to be re-uploaded to remote HPC facilities to re-execute simulations. With Meshtool these costs can be fully avoided.

Meshtool has been adopted quickly beyond our lab by leading labs within the cardiac modeling community. As Meshtool is shipped as an integral part of our *in silico* modeling platform comprising the CARPentry simulator and *carputils* as a platform for definition and execution of *in silico* experiments Meshtool is used by the entire community using our modeling ecosystem. Based on download metrics and the user mailing lists there are about 120 users in about ten labs world wide, with about sixty highly active users producing around twenty cardiac modeling research papers per year. Beyond the cardiac modeling community Meshtool we are not aware of any academic use of Meshtool. However, Meshtool can be equally useful in any image-based modeling studies in other areas of biological research where anatomical and functional features are derived from tomographic images such as modeling other organs such as e.g. brain, lungs or liver.

Meshtool is available under an open source license and can be freely used in commercial applications. There are no plans to market Meshtool as a standalone software, but it has been integrated and is being used in workflows of commercial cardiac modeling endeavors.

## 5 Conclusions

Meshtool is software for mesh generation and manipulation that is tailored for automating complex mesh manipulation tasks in image-based modeling building and functionalization work-flows. Meshtool has been primarily designed for advanced cardiac modeling studies that heavily rely on the ability to generate and functionalize a larger number of individualized *in silico* models to build virtual cohorts that capture the vast variability of cardiac anatomy in terms of size, shape and morphology. Integrating Meshtool in modeling pipelines as used in the cardiac modeling community significantly increases efficiency through automation to keep processing tractable, reduces costs in terms of time and man power needed, and improves quality of models by minimizing inevitable errors due to manual mesh manipulation.

## Figures and Tables

**Table 1:** The most important modes of Meshtool, grouped by category.

Category	Modes
sub-mesh management	extract mesh, insert submesh, map, restore mapping
data mapping	extract data, insert data
mesh and data manipulation	smooth mesh, smooth data, interpolate, merge meshes
mesh format conversion	convert
surface extraction	extract surface
re-meshing	resample mesh, generate mesh, merge surface
image stack manipulation	itk
mesh quality control	clean quality
mesh info and statistics	query

## Footnotes

Code metadata

**Table:**

Current code version	v15
Permanent link to code/repository used for this code version	https://github.com/ElsevierSoftwareX/SOFTX_2019_291
Legal Code License	GPLv3
Code versioning system used	git
Software code languages, tools, and services used	C_++_, OpenMP, make
Compilation requirements, operating environments & dependencies	GNU, Intel or clang C_++_compiler with 2011 standard support, GNU make
If available Link to developer documentation/manual	https://bitbucket.org/aneic/meshtool/src/master/README.md
Support email for questions	aurel.neic@numericor.at
